# Placebo and nocebo effects of percutaneous needle electrolysis and dry-needling: an intra and inter-treatment sessions analysis of a three-arm randomized double-blinded controlled trial in patients with patellar tendinopathy

**DOI:** 10.3389/fmed.2024.1381515

**Published:** 2024-06-06

**Authors:** Víctor Doménech-García, Daniel Pecos-Martín, Julia Blasco-Abadía, Pablo Bellosta-López, María Pilar López-Royo

**Affiliations:** ^1^Facultad de Ciencias de la Salud, Universidad San Jorge, Zaragoza, Spain; ^2^Departamento de Enfermería y Fisioterapia, Universidad de Alcalá, Alcalá de Henares, Spain

**Keywords:** placebo, nocebo, needling techniques, percutaneous needle electrolysis, dry needling, tendinopathy

## Abstract

**Objective:**

This study aimed to investigate the influence of potential placebo and nocebo effects on pain perception of percutaneous needle electrolysis (PNE) in individuals with patellar tendinopathy.

**Methods:**

In this three-arm randomized double-blinded controlled trial, intra and inter-session pain perception data from 48 sporting participants with patellar tendinopathy between 18 and 45 years were investigated. Participants were divided into 3 parallel groups: “no-sham group” [PNE intervention], “single-sham group” [sham PNE by using dry needling], and “double-sham group” [sham PNE by using sham needles]. Every group received 4 sessions of the needling therapies targeting the patellar tendon over 8 weeks and was instructed to perform a unilateral eccentric exercise program of the quadriceps muscle on the affected side. Clinical and needle-related pain was assessed before, during, and after each treatment session using a visual analog scale.

**Results:**

No differences were found between groups intra- or inter-session in terms of pain reduction (*P* = 0.424) despite clinical pain decreased in all groups since the first treatment session (*P* < 0.001). Furthermore, although the double-sham group showed a lower percentage of participants reporting needle-related pain during needle intervention (*P* = 0.005), the needle-related pain intensity after needle intervention was similar between groups (*P* = 0.682). Moreover, there were no group differences for the duration of pain sensation after any needle intervention (*P* = 0.184), extending in many cases beyond 24 h.

**Conclusion:**

Needling therapies for individuals with patellar tendinopathy are prone to elicit placebo effects regarding clinical pain and nocebo effects regarding needling-related pain. Clinicians and physical therapists treating musculoskeletal pain conditions should consider the added value and potential mechanisms of action before routinely using needle techniques.

## 1 Introduction

Musculoskeletal painful disorders represent the primary contributor to rehabilitation needs and a major worldwide health problem (1). Patients with musculoskeletal painful disorders often seek a physiotherapist for assistance, and physiotherapists employ various therapeutic interventions, which are often complex, to reduce pain and disability (2). Ideally, specific treatment effects drive most of these changes, although non-specific effects such as regression to the mean, natural history, and contextual effects also contribute (3). Placebo effects can be described as beneficial effects that are not due to an active treatment components (4). and depends on contextual factors related to the reduction of symptoms caused by the psychosocial context, such as positive expectations or patient satisfaction, and not solely by the properties of the treatment itself (5). In contrast, nocebo effects are adverse treatment outcomes elicited by non-active treatment components (4) and are produced by negative expectations or context that may exacerbate the patient’s symptoms (6).

Clinically, placebo and nocebo effects are important during therapy administration, representing the result of the adjuvant or harmful use of contextual factors (7, 8). Information provided about treatment, patient expectations, previous encounters with a procedure, therapist characteristics, and the therapeutic relationship between the patient and the therapist can all generate these effects (8–13). While isolated placebo treatments seem to lack clinical meaningfulness, recent meta-analysis findings show that within the realm of non-pharmacological conservative interventions for musculoskeletal conditions, the placebo effect can contribute up to 30% of the minimally clinically important difference (14). Interestingly, the authors hypothesized that certain interventions such as needles or manual therapy may elicit even more substantial placebo effects. However, despite adequately-designed randomized controlled clinical trials that should include placebo controls to disentangle placebo and nocebo effects from the general effect of the intervention (15), only a very small proportion of randomized controlled clinical trials testing physiotherapy interventions do so (16).

In recent years, minimally invasive procedures for managing musculoskeletal painful disorders, such as dry needling (DN) or percutaneous needle electrolysis (PNE), have gained global popularity (17, 18). PNE is an invasive approach that involves applying a galvanic current through an acupuncture needle into the soft tissue lesion to elicit a local inflammatory response (19). Discomfort related to applying galvanic current could make PNE an unpleasant procedure for the patient. Indeed, the most common adverse effects of PNE, such as pain during the intervention and in the days following treatment, (18) are similar to those observed in DN (20). Furthermore, using needles as a therapeutic tool may cause a certain degree of apprehension in the patient, (20) and fear of needles or fear of pain could predispose the subject to react with negative emotions to pain and in anticipation of pain (21, 22). Conversely, it is unknown to what extent these interventions produce improvements in patients with musculoskeletal painful disorders due to non-specific effects such as placebo hypoalgesia. Placebo hypoalgesia is observed when a sham intervention results in pain relief and can also be acquired through operant conditioning. This uncertainty is probably due to the challenge that represents its evaluation represents, not only in needling interventions but in musculoskeletal interventions in general (15). Although the PNE technique has been compared with placebo interventions, the potential influence of the placebo effect on the results has not been considered (23).

Therefore, this study aimed to investigate the influence of potential placebo and nocebo effects on pain perception of an intratissue PNE-based intervention in individuals with patellar tendinopathy.

## 2 Material and methods

### 2.1 Study design and settings

This study was part of a three-arm randomized double-blinded controlled trial (ClinicalTrial.gov: NCT02498795) (24). The study followed the Helsinki Declaration and was approved by the local Ethics Committee (C.P.−C.I. PI15/0017), and all participants consented their enrolling in this study.

### 2.2 Participants

Adults aged between 18 and 45 years were recruited from various sports clubs and federations. To be eligible, participants had to meet specific inclusion criteria: (1) experienced anterior knee pain below the patella while engaging in sports for over 3 months; (2) engaged in sports activities at least 3 times a week; and (3) scored below 80 on the Victorian Institute of Sport Assessment-Patellar questionnaire (VISA-p). Exclusion criteria included: (1) knee surgery in the past 6 months; (2) patellar tendon corticosteroid injection in the past 3 months; (3) diagnosed with chronic joint disease; (4) contraindications for needling (e.g., needle phobia, needle material allergy); (5) consumption of anti-inflammatory, analgesic, or antibiotic medications within the past 48 h; and (6) undergoing concurrent physiotherapy treatment. Additionally, ultrasound examination of the knee joint and adjacent musculoskeletal structures was conducted before enrollment in the study. This examination aimed to exclude the presence of joint effusion or signs of inflammation and to identify the presence of degenerative signs, characterized by a hypoechoic area in the body of the tendon. None of the participants in the final selected sample had received prior needling treatment in the tendon.

### 2.3 Groups and interventions

**FIGURE 1:**
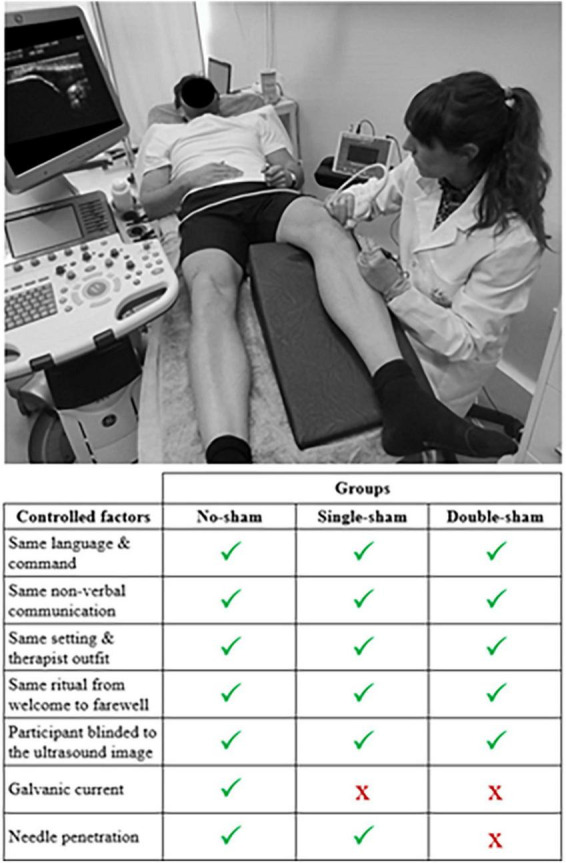
Illustration depicting the common setting and intervention procedures for all three groups, along with a list of controlled factors.

Participants were divided into three groups according to the intervention received: (1) PNE [no-sham group], (2) sham PNE by using DN [single-sham group], and (3) sham PNE by using sham needles [double-sham group]. The setting and procedure followed with each participant was similar regardless of group, isolating the effects related to galvanic current and needling (Figure 1). All interventions were targeted at the patellar tendon.

#### 2.3.1 No-sham group

For the no-sham group, 0.25 × 0.25 mm needles (*APS safety tube dry needles; Agupunt*) were connected to the electrolysis device (*model EPI^®^, CESMAR Electromedicina. S.L., Spain*). The researcher utilized ultrasonography to guide the procedure, ensuring precise application to the injured area and maintaining safety. The needle was inserted into the injured area three times, targeting the hypoechoic region within the patellar tendon. Each insertion lasted 3 s and involved the use of 3 mA of galvanic current.

#### 2.3.2 Single-sham group

In the single-sham group, the needle was inserted following the same protocol as in the no-sham group. The only difference was that the electrolysis device was turned on with a current intensity of 0 mA (i.e., no galvanic current). The needle reached the relevant treatment area in the patellar tendon guided by ultrasonography.

#### 2.3.3 Double-sham group

For the double-sham group, a sham needle was placed upon the treatment zone, simulating the same procedure as in the other groups. In addition, the needle was manipulated in and out to simulate a real treatment. The holder had a cover over the bottom part to prevent the needle from contacting the skin.

### 2.4 Procedure

Participants underwent 4 intervention sessions over an 8-week period, with each session spaced 2 weeks apart. During each session, a standardized procedure was followed to ensure participant blinding during needle interventions, attempting to overcome biases found in previous studies on needling techniques (25). Participants were positioned supine with their knee flexed at 20°, supported by a pillow. The researcher performing the intervention wore latex gloves and cleansed the area with a 70% propan-2-ol antiseptic solution. A disposable protective cover was applied to a lubricated ultrasound probe (*Logic S7 Expert, General Electric Healthcare*), which was used for real-time ultrasound guidance. The ultrasound display screen was positioned behind the participants. Each participant held the anode connected to the electrolysis device and received the following instruction: “During the needle intervention, please try to remain still. The treatment may cause some pain or discomfort. If you experience any, please let me know, and I will stop immediately.” After needle removal, the area was gently compressed with cotton wool for 5 s (Figure 1).

At the end of the study, participants were asked via email to guess the type of treatment they received. The options provided were: “No needling treatment,” “Needling treatment,” or “I don’t know.” If participants selected “Needling treatment,” they were further asked to specify whether they received PNE or DN, with the options being “PNE,” “DN,” or “I don’t know.”

Complementary to the real or sham needle intervention, all participants were instructed to perform a unilateral eccentric exercise program of the quadriceps muscle on the affected side, specifically aimed at the patellar tendon. This program consisted of performing 3 sets of 15 repetitions daily on a decline board (26). The correct execution of the exercise as well as the follow-up of the prescribed program was monitored by the research team every two weeks, coinciding with the day the participant received the intervention.

### 2.5 Randomization

Participants who fulfilled the inclusion criteria and consented to participate in the trial were randomly assigned by a researcher not involved in the study by generating random participant sequences with a 1:1:1 allocation using an opaque envelope, with a block size of 15 participants, using a computer program (Randomizer).^1^

### 2.6 Outcome measures

Assessments were made by an assessor blinded to group allocation. The primary outcomes for placebo and nocebo effects were clinical pain reduction after needle intervention and needle-related pain intensity after needle intervention, respectively. While the secondary outcomes were clinical pain intensity before needle intervention, clinical pain intensity during a provocative test after needle intervention, needle-related pain during needle intervention, and days until needle-related pain sensation completely disappears after needle intervention. Outcome measures in this study are detailed in Table 1. Description and clinical findings in VISA-p and ultrasonographic measures after the 8-week period are available elsewhere (27).

**TABLE 1:** Detailed description of the outcome variables included in the study.

Variable name	Variable description
Clinical pain intensity before needle intervention.	Participants rated their pain before each needle intervention using a Visual Analogue Scale where a score of 0 indicated “absence of pain”, whereas a score of 10 represented the “maximum tolerable pain”.
Needle-related pain during needle intervention.	Participants were asked to report by “yes” or “no” if they had pain during the needle intervention (i.e., Have you felt any pain during the procedure?).
Needle-related pain intensity after needle intervention.	Participants rated their level of pain in the tendon area after each needle intervention (i.e., How painful is the area of the puncture after the procedure?) by using a Visual Analogue Scale where a score of 0 indicated “absence of pain”, whereas a score of 10 represented the “maximum tolerable pain”.
Clinical pain intensity during a provocative test after needle intervention.	Five minutes after the real or sham needle intervention, participants performed a provocative test for the patellar tendon, consisting of a half single squat (until 90 degrees of knee flexion) performed with the symptomatic leg. Upon completion, participants were asked to rate their pain intensity during the provocative test using a Visual Analogue Scale where a score of 0 indicated “absence of pain” whereas a score of 10 represented the “maximum tolerable pain”.
Clinical pain reduction after needle intervention.	Pain reduction after needle intervention was calculated by subtracting pain intensity values before needle intervention from pain intensity values during the provocative test.
Days until needle-related pain sensation completely disappears after needle intervention.	At the beginning of each intervention session, except for the first session, the participants reported the duration of pain sensation after the last intervention (i.e., How long was the area of the puncture painful after the last session?), where the possible answers were: “No pain or soreness”, “ < 24 h”, “24–48 h”, or “ > 48 h”.

### 2.7 Statistical analysis

Statistical analysis was performed using SPSS, v.25 (IBM Corp., Chicago, IL). A *P*-value < 0.05 was accepted as a significant difference between compared variables. Variables distribution was assessed using the Shapiro-Wilk test and described in percentage, mean and standard deviation or median and interquartile range, according to the distribution of data. Differences between groups were compared using chi-squared tests (χ^2^) for categorical data and mixed-model repeated-measures analysis of variance (RM-ANOVA) for continuous data with *time* (session 1, 2, 3, and 4) as within and *group* (no-sham group, single-sham group, and double-sham group) as between factors. Pairwise Bonferroni comparisons were performed as post hoc analyses.

### 2.8 Sample size calculation

The original sample size was calculated based on the VISA-p, and details were reported elsewhere (27). However, a secondary post hoc sample size calculation with G*Power (v3.1.9.2, Heinrich-Heine-University, Dusseldorf, Germany) revealed the feasible sample size for a mixed model RM-ANOVA with three groups (no-sham group, single-sham group, and double-sham group) participating in four experimental sessions. With a power of 90% and an alpha level of 0.01, a total of 42 participants (14 per group) were needed for participation to detect the minimal important difference of 1.2 points (partial *η*^2^ = 0.05) in the Visual Analogue Scale (28).

## 3 Results

**FIGURE 2:**
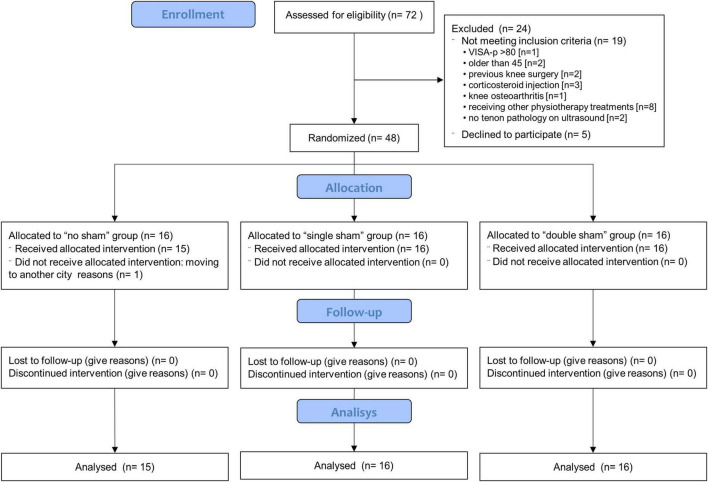
Participant flow chart. DN, dry needling group; PNE, Percutaneous needle electrolysis group.

Recruitment began in January 2019 and was completed in December 2019. Out of the 72 subjects assessed for eligibility, 5 declined to participate, while 19 did not meet eligibility criteria (24). A total of 48 participants (16 per group) were enrolled and received the allocated intervention. One participant in the no-sham group withdrew after the first session due to moving to another city and was subsequently removed from the statistical analysis. Figure 2 shows the study flowchart.

Table 2 shows the characteristics of the 47 participants who completed the study. No significant differences were found between the groups regarding sociodemographic and clinical variables at baseline. Participants who completed the blinding questionnaire (*n* = 29; no-sham group: 12; single-sham group: 9, and double-sham group: 8) reported receiving a needle intervention, from which 82% (*n* = 23) indicated PNE as the needle intervention.

**TABLE 2:** Participant characteristics in the three study groups.

	No sham (*n* = 15)	Single sham (*n* = 16)	Double sham (*n* = 16)	*P* value
Age, years	36.0 [28.0–39.5]	33.0 [31.0–43.5]	36.5 [31.2–39.0]	0.57
Women, *n* (%)	2 (13.3%)	3 (18.8%)	1 (6.3%)	0.57
Weight, kg	80.0 [66.9–89.9]	75.5 [62.5–83.3]	80.0 [73.2–84.0]	0.50
Height, cm	179 [173–181]	176 [168–185]	179 [176–182]	0.83
BMI, kg/m^2^	24.4 [22.7–27.1]	23.9 [21.7–26.2]	24.9 [23.0–26.6]	0.32
Sport activity, times/week	4 [4–5]	5 [4–7]	5 [4–7]	0.19
Symptoms duration, months	15.0 [12.0–24.0]	10.5 [4.3–18.0]	10.5 [6.0–23.0]	0.98
VISA-P	48.9 [30.0–75.0]	57.2 [29.0–79.0]	55.5 [42.0–79.0]	0.16

Median [interquartile range: 25th-75th percentiles]. BMI, Body Mass Index; VISA-P, Victorian Institute of Sports Assessment Questionnaire, patellar tendon.

### 3.1 Clinical pain intensity before needle intervention

No *time* and *group* interaction (RM-ANOVA: *F*_6,132_ = 1.1; *P* = 0.357) was found for pain intensity before needle intervention, indicating no differences in the evolution of pain intensity between groups across sessions. However, a *time* effect was found (RM-ANOVA: *F*_3,132_ = 16.5; *P* < 0.001), indicating that the three groups decreased pain intensity over the sessions. Post-hoc analysis showed a lower pain intensity at the beginning of session 4 compared to session one in all groups (no-sham group: *P* < 0.001; single-sham group: *P* = 0.011, and double-sham group: *P* = 0.037). See Table 3.

**TABLE 3:** Outcome measures in the three study groups.

	No sham (*n* = 15)	Single sham (*n* = 16)	Double sham (*n* = 16)	*P* value
Clinical pain intensity before needle intervention (VAS 0–10)				0.357^§^
Session 1	5.1 ± 1.6	4.0 ± 1.8	5.0 ± 2.1	
Session 2	4.5 ± 2.2	3.0 ± 1.5	4.3 ± 2.0	
Session 3	3.6 ± 1.6	3.1 ± 2.2	4.5 ± 2.5	
Session 4	2.6 ± 1.7	2.3 ± 1.9	3.5 ± 2.1	
**Needle-related pain during needle intervention (n yes, %)**				
Session 1	12 (80%)*	14 (88%)*	4 (25%)	< 0.001^#^
Session 2	12 (80%)*	16 (100%)*	6 (38%)	< 0.001^#^
Session 3	12 (80%)*	15 (94%)*	7 (44%)	0.005^#^
Session 4	12 (80%)*	13 (81%)*	4 (25%)	< 0.001^#^
Needle-related pain intensity immediately after needle intervention (VAS 0–10)				0.682^§^
Session 1	1.7 ± 1.9	1.7 ± 1.9	1.1 ± 2.1	
Session 2	2.0 ± 2.5	2.1 ± 2.4	1.3 ± 2.0	
Session 3	1.9 ± 2.3	1.3 ± 2.1	1.3 ± 1.9	
Session 4	1.3 ± 2.2	1.5 ± 1.8	0.8 ± 1.6	
Clinical pain intensity during a provocative test after needle intervention (VAS 0–10)				0.937^§^
Session 1	0.0 ± 0.1	0.7 ± 1.4	0.9 ± 1.5	
Session 2	0.5 ± 1.8	0.9 ± 1.7	0.8 ± 1.6	
Session 3	0.3 ± 1.0	0.3 ± 1.0	0.5 ± 1.4	
Session 4	0.0 ± 0.0	0.3 ± 1.0	0.4 ± 1.2	
**Duration of pain sensation after needle intervention (n, %)**				
Session 1				0.284^#^
No pain	1 (7%)	0 (0%)	0 (0%)	
< 24 h	8 (53%)	11 (69%)	8 (50%)	
24–48 h	6 (40%)	3 (19%)	8 (50%)	
> 48 h	0 (0%)	2 (13%)	0 (0%)	
Session 2				0.948^#^
No pain	2 (13%)	2 (13%)	3 (19%)	
< 24 h	9 (60%)	8 (50%)	8 (50%)	
24–48 h	4 (27%)	6 (38%)	5 (31%)	
> 48 h	0 (0%)	0 (0%)	0 (0%)	
Session 3				0.670^#^
No pain	2 (13%)	2 (13%)	3 (19%)	
< 24 h	8 (53%)	12 (75%)	8 (50%)	
24–48 h	3 (20%)	2 (13%)	4 (25%)	
> 48 h	2 (13%)	0 (0%)	1 (6%)	

Mean ± SD. VAS, Visual Analogue Scale.

^§^*P* value after RM-ANOVA.

^#^*P* value after chi-squared test.

*P < 0.05 indicating a higher frequency compared to the double sham group.

### 3.2 Needle-related pain during needle intervention

Significant differences existed between the frequencies of participants reporting pain in the three groups during all the needle interventions (*χ*^2^ ≥ 10.6; *P* ≤ 0.005). Specifically, 80% of the no-sham group, 81% to 100% of the single-sham group, and 25% to 44% of the double-sham group reported pain during needle intervention. See Table 3.

### 3.3 Needle-related pain intensity immediately after needle intervention

No *time* and *group* interaction (RM-ANOVA: *F*_6,132_ = 0.7; *P* = 0.682) or *time* effect (RM-ANOVA: *F*_3,132_ = 1.7; *P* = 0.168) was found for pain intensity after needle intervention, indicating that the painful sensation after needle intervention was similar between groups in all sessions. See Table 3.

### 3.4 Clinical pain intensity during a provocative test after needle intervention

No *time* and *group* interaction (RM-ANOVA: *F*_6,132_ = 0.4; *P* = 0.937) or *time* effect (RM-ANOVA: *F*_3,132_ = 1.7; *P* = 0.179) was found for painful sensation after needle intervention, indicating that the painful sensation after needle intervention was similar between groups in all sessions. See Table 3.

### 3.5 Clinical pain reduction after needle intervention

**FIGURE 3:**
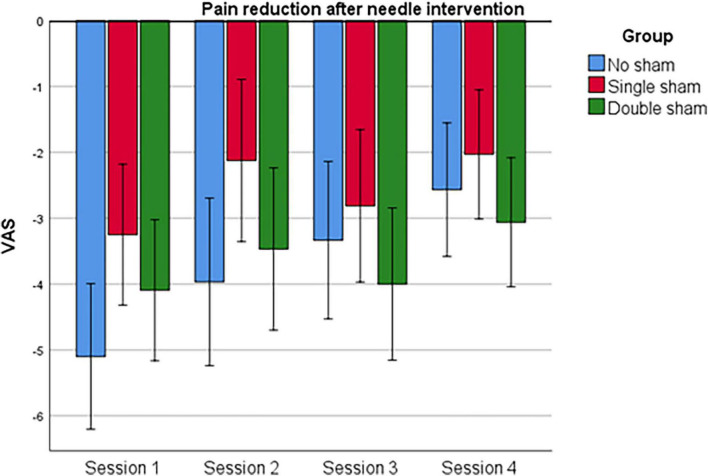
Clinical pain (tendon pain) reduction after the intervention.

No *time* and *group* interaction (RM-ANOVA: *F*_6,132_ = 1.0; *P* = 0.424) was found for pain reduction after needle intervention, indicating no differences in the evolution of pain reduction between groups across sessions. However, a *time* effect was found (RM-ANOVA: *F*_3,132_ = 6.3; *P* < 0.001), indicating that pain reduction within the three groups differed between sessions. *Post hoc* analysis showed a lower pain reduction at session 2 (*P* = 0.027) and session 4 (*P* = 0.001), compared to session 1 in all groups. See Figure 3.

### 3.6 Days until needle-related pain sensation completely disappears after needle intervention

There were no differences between the frequencies of participants reporting the duration of pain sensation after any needle intervention (*χ*^2^ ≤ 8.8; *P* ≥ 0.184). See Table 3.

### 3.7 Adverse effects

No adverse events were reported other than the previously described needle-related pain itself and the post-puncture pain described above, which were part of the outcome measures.

## 4 Discussion

This study is the first to investigate placebo and nocebo effects potentially associated with an invasive needle technique by implementing single-sham and double-sham procedures in individuals with patellar painful tendinopathy. The findings suggest that needling techniques, specifically PNE, induce significant placebo and nocebo effects on both clinical and needle-related pain, respectively.

### 4.1 Contextual effects in clinical tendon pain

The three groups similarly improving their pain over time, both at rest (before and after intervention) and during a functional provocative test, might reflect different effects. On the one hand, these findings might only indicate a direct positive effect of eccentric exercise on tendon pain, as previously reported (29). Furthermore, it is unlikely that there was no effect at all from the PNE intervention on pain, as previous studies have found a pain reduction effect in tendinopathies (18). Therefore, it is unlikely that eccentric exercise explains the clinical pain improvements observed in the three groups.

On the other hand, the persistence of patellar tendinopathy symptoms for 18 months in the present study, consistent with previous literature, (30) suggests that non-specific effects such as regression to the mean or natural history might partially explain the observed improvements in clinical pain. However, both the present study and previous literature show a high standard deviation in the duration of symptoms, either at the mean value or above. This substantial variability makes regression to the mean and natural history less likely to account for the clinical pain improvement observed in the three groups. Moreover, it is important to note that these non-specific factors would not explain the observed nocebo effect in needle-related pain. Therefore, it can be inferred that non-specific effects, such as placebo and nocebo responses, are at least partially responsible for the reduction in pain among patients with patellar tendinopathy undergoing needling therapies.

A recent study has shown that expectations may not play as significant role as prior therapeutic experiences in placebo and nocebo responses in healthy individuals (31). Although the connection between these non-specific effects and the psychological characteristics of the participants remains unknown, a recent study with high methodological quality and one of the largest samples in placebo research has shown that individuals with chronic pain benefiting from placebo are characterized by low emotional stress, pain-related fear, and catastrophizing (5). However, catastrophizing has been linked to patellar tendinopathy only in individuals with more severe symptoms (32). The present study, based on the baseline pain VAS values of participants, indicates that in most cases, tendinopathies were moderate to low. Therefore, it can be hypothesized that the sample in the current study did not have a high psychological burden and were more likely to benefit from placebo effects.

### 4.2 Contextual effects in needling-related pain

As expected, most participants reported pain during the intervention when a needle was inserted into the tendon. Interestingly, up to 44% of participants in the double-sham group also reported pain during intervention. Furthermore, all groups reported the same amount of needle-related pain immediately after the intervention, and up to 25% of participants in the double-sham group continued experiencing pain even 24-48h after the intervention. Overall, these findings indicate that needling interventions are susceptible to non-specific effects, aligning with previous literature (33).

Although not measured on participants, subjective factors such as expectations may have modulated pain (34) after the needling intervention (35). Increased pain due to the expectation of more pain following an intervention is a nocebo effect observed previously (36, 37). A study in patients with chronic shoulder pain found increased mechanical hyperalgesia immediately following sham dry needling that lasted for 24h in individuals with shoulder pain. The study attributed these changes to a nocebo effect generated by negative expectations associated with the instructions given before the procedure (38). In addition to negative expectations, other factors that might have mediated a nocebo effect in post needling-related pain include classical conditioning, observational learning, (39) and prior therapeutic experiences (31).

### 4.3 Clinical implications

These findings clearly reflect that needling interventions in individuals with patellar tendinopathy involve intrinsic positive and negative non-specific factors. Therefore, the contextual factors of needling therapies can trigger both placebo and nocebo responses and must be considered in the clinical context. This serves as a general recommendation for physiotherapists and musculoskeletal clinicians, who are encouraged to understand and manage the contextual factors [e.g., patients’ expectations, past treatments, verbal suggestions (40)] that enhance placebo effects and avoid nocebo effects (2, 41).

In this context, it has been shown that individuals with low back pain who received an intense briefing on the adverse side effects of acupuncture tended to exhibit a higher adverse side effect score compared to participants who received a regular adverse side effect briefing (42). However, other studies did not find a significant effect of patient expectations on the short-term effects of DN on pain intensity and pain-inducing stimulus intensities in people with neck pain; (43) or showed that DN treatment can produce beneficial effects on neck pain and tissue mechanosensitivity, regardless of whether participants received a positive, negative, or neutral verbal stimulus before treatment (44).

All participants in the current study experienced post-DN soreness that did not influence treatment outcomes. Interestingly, participants were not very concerned about post-needling soreness as long as their clinical pain complaint decreased. Therefore, it seems that briefings about treatment in routine care might not be as important as previously thought (42). Other variables, such as previous therapeutic experiences (31) or patient satisfaction, might also be important for clinically managing placebo and nocebo effects, as these are factors that can impact the outcome of rehabilitation (45). Indeed, according to several double-blind trials testing pain treatments, the placebo effect can be similar to specific treatment effects (9). In summary, current research on placebo effects indicates that the ethical enhancement of a placebo without using placebos or misinforming patients is feasible and ethical.

### 4.4 Limitations

This study did not control for demand characteristics, (46) potentially influencing results as participants may have guessed evaluator’s expectations. Including measures of psychological factors such as fear, emotional stress, catastrophizing, and participants’ satisfaction or subjective perception of improvement as indirect measures of the expectations, would have strengthened the study design. This would have allowed ascribing the consistent placebo and nocebo effects found to specific contextual factors. Future studies should explore placebo and nocebo effects in other needle-based techniques for the treatment of musculoskeletal pain.

## 5 Conclusion

The results of this research show that needling therapies for individuals with patellar tendinopathy are prone to elicit placebo and nocebo effects regarding clinical and needling-related pain, respectively. Future studies involving participants with musculoskeletal painful disorders should incorporate placebo-controlled designs and monitor both clinical and needling-related pain during the treatment period. Additionally, given the clear presence of these effects, physiotherapists and musculoskeletal clinicians are encouraged to delve into the knowledge of placebo and nocebo to better manage their patients with tendinopathies.

## Data availability statement

The original contributions presented in this study are included in this article/Supplementary material, further inquiries can be directed to the corresponding author.

## Ethics statement

The studies involving humans were approved by the Comité de Ética de la Investigación de la Comunidad Autónoma de Aragón (C.P.−C.I. PI15/0017). The studies were conducted in accordance with the local legislation and institutional requirements. The participants provided their written informed consent to participate in this study. Written informed consent was obtained from the individual(s) for the publication of any potentially identifiable images or data included in this article.

## Author contributions

VD-G: Conceptualization, Funding acquisition, Investigation, Resources, Writing−original draft, Writing−review and editing. DP-M: Validation, Writing−original draft, Writing−review and editing. JB-A: Conceptualization, Investigation, Visualization, Writing−review and editing. PB-L: Conceptualization, Data curation, Formal analysis, Investigation, Methodology, Software, Supervision, Validation, Writing−original draft, Writing−review and editing. ML-P: Conceptualization, Data curation, Investigation, Methodology, Project administration, Resources, Supervision, Writing−original draft, Writing−review and editing.

## Footnotes

1
https://www.randomizer.org
