# Correction: Placebo and nocebo effects of percutaneous needle electrolysis and dry-needling: an intra and inter-treatment sessions analysis of a three-arm randomized double-blinded controlled trial in patients with patellar tendinopathy

**DOI:** 10.3389/fmed.2026.1851424

**Published:** 2026-04-28

**Authors:** Víctor Doménech-García, Daniel Pecos-Martín, Julia Blasco-Abadía, Pablo Bellosta-López, María Pilar López-Royo

**Affiliations:** 1 Facultad de Ciencias de la Salud, Universidad San Jorge, Zaragoza, Spain; 2 Departamento de Enfermería y Fisioterapia, Universidad de Alcalá, Alcalá de Henares, Spain

**Keywords:** placebo, nocebo, needling techniques, percutaneous needle electrolysis, dry needling, tendinopathy

In the published article, there was an error in the description of the study design. The term “secondary analysis” was incorrectly used in the Abstract section. The correct description is that the study used data from a previously conducted randomized controlled trial, focusing on pain-related variables that had not been analyzed in prior publications from the original trial.

In the abstract, the sentence previously read:

“Methods: In this secondary analysis of a three-arm randomized double-blinded controlled trial, intra and inter-session pain perception data from 48 sporting participants with patellar tendinopathy between 18 and 45 years were investigated.”

The corrected sentence appears below:

“**Methods:** In this three-arm randomized double-blinded controlled trial, intra and inter-session pain perception data from 48 sporting participants with patellar tendinopathy between 18 and 45 years were investigated.”

The original version of this article has been updated.

